# *Siccibacter turicensis* from Kangaroo Scats: Possible Implication in Cellulose Digestion

**DOI:** 10.3390/microorganisms8050635

**Published:** 2020-04-27

**Authors:** Sudip Dhakal, Jarryd M. Boath, Thi Thu Hao Van, Robert J. Moore, Ian G. Macreadie

**Affiliations:** School of Science, RMIT University, Bundoora, Victoria 3083, Australia; sudip.dhakal@rmit.edu.au (S.D.); jarrydboath@gmail.com (J.M.B.); thithuhao.van@rmit.edu.au (T.T.H.V.); rob.moore@rmit.edu.au (R.J.M.)

**Keywords:** cellulase, *Enterobacteriaceae*, gut microbiota, kangaroo nutrition, scat microbe

## Abstract

Microbiota in the kangaroo gut degrade cellulose, contributing to the kangaroo’s energy and survival. In this preliminary study, to discover more about the gut microbes that contribute to the survival of kangaroos, cellulose-degrading bacteria were isolated from kangaroo scats by selection on solidified media containing carboxymethyl cellulose as the main carbon source. One frequently occurring aerobic bacterium was *Siccibacter turicensis*, a microbe previously isolated in fruit powder and from a patient with angular cheilitis. The whole genome sequence of the kangaroo isolate was obtained using the Illumina MiSeq platform. Its sequence shared 97.98% identity of the *S. turicensis* Type strain, and the ability of the Type strain to degrade cellulose was confirmed. Analysis of the genomic data focused on the cellulose operon. In addition to genes from the operon, we suggest that a gene following the operon may have an important role in regulating cellulose metabolism by signal transduction. This is the first report of *S. turicensis* found within microbiota of the animal gut. Because of its frequent presence in the kangaroo gut, we suggest that *S. turicensis* plays a role in cellulose digestion for kangaroos.

## 1. Introduction

Animals are devoid of enzymes capable of degrading cellulose, however, intestinal microbiota perform the task of enabling access to nutrients and energy sources that would be otherwise unavailable [1]. The microbes capable of performing this task are currently uncharacterised, however, metagenomics studies provide information on many of the microbes that are present and significant clues on what their roles may be. However, little work has been done on culturing these microbes for assays and characterization: most are currently unculturable, and many are obligate or facultative anaerobes. Kangaroos have a diet comprising grass only, so it is expected that they are highly dependent on gut microbes specialised in the digestion of grass [2].

Cellulose degrading enzymes are often used for various industrial purposes. Recent studies have shown the effects of cellulases, obtained from the *Bacillus subtilis* strain BY3, as an antibacterial agent and for biofuel production from agricultural wastes [3]. *Acinetobacter* spp. are established as effective degraders of similar cellulosic wastes for the production of bioethanol, which may have a crucial role in fulfilling current and future energy demands [4]. In some recent studies, cellulases have been evaluated successfully for decontaminating the cellulosic biofilm-forming microbes like *Salmonella* spp., when used in processing vegetables and meat [5,6]. In a similar study, cellulases have been used to improve the feedstock for broiler chickens [7]. Moreover, diverse types of fungi and bacteria have been found to produce the enzymes for degradation of the cellulose that can provide renewable energy [4,8,9,10,11].

For this preliminary investigation of microorganisms capable of cellulose degradation, fresh scats of wild kangaroos were used to obtain microbes growing aerobically on solidified media containing carboxymethyl cellulose (CMC) plates. The most abundant microbe to be isolated was *Siccibacter turicensis*. Most isolates possessed a relatively weak cellulose-degrading activity as judged by very small zones when clearing CMC. The whole genome of kangaroo-associated *S. turicensis* was analysed to verify the similarity to the Type strain, and to provide bioinformatic information about cellulose degradation in this microorganism. This analysis indicates that the cellulase activity is encoded within the *bcs* operon, first identified in *Acetobacter xylinum* [12]. The *bcs* operon is composed of nine genes involved in cellulose synthesis and degradation [13].

## 2. Materials and Methods

### 2.1. Isolation of Cellulose Degraders 

Fresh scats of free-roaming Eastern Grey Kangaroos from Warrandyte, Victoria, Australia, were collected and used for culture on the same day. Samples were plated onto nutrient agar, and microorganisms utilising cellulose as a carbon source were selected on CMC plates [14]. Isolates of *S. turicensis* from multiple kangaroos were obtained over multiple years, and the identity of all isolates were analysed using 16S rRNA gene sequencing.

The *S. turicensis* DSM 18397 was purchased from the German Culture Collection, DSMZ.

### 2.2. MALDI-TOF Mass Spectroscopy Analysis

Cells for matrix-assisted laser desorption ionization time-of-flight (MALDI-TOF) mass spectroscopy (MS) analysis were prepared and analysed using a Bruker Biotyper (Preston, Vic., Australia) [15]. The *S. turicensis* mass spectrum was added to the RMIT University Bruker library for rapid identification of *S. turicensis*.

### 2.3. Partial 16S rRNA Gene Sequencing 

For rapid molecular characterization of isolates, partial 16S rRNA gene sequences were obtained. Bacteria were boiled in water and an aliquot was subjected to PCR, utilising the universal primers CCAGACTCCTACGGGAGGCAGC and CTTGTGCGGGCCCCCGTCAATTC, to amplify a conserved portion of the 16S rRNA gene [16]. The 0.6 kb PCR product was purified and submitted to the Micromon sequencing facility at Monash University, Victoria, Australia. The sequence was used for identification of isolates using BLASTN searches. 

### 2.4. Whole Genome Sequencing and Gene Annotation

The whole genome of one isolate, #493, was sequenced using an Illumina MiSeq sequencer. The whole genome was assembled using A5-miseq, which uses reads from the sequencer and read pairing information for contig generation [17]. The whole genome sequence has been submitted to National Center for Biotechnology Information (NCBI) database and has the accession number RYYT00000000.

The genes and RNA sequences from the whole genome sequence were annotated using rapid annotations using subsystems technology (RAST), the local RAST toolkit (myRAST) [18]. Sequences were analysed to identify the bacterium and genes likely to encode proteins involved in cellulose metabolism.

### 2.5. Identification of the Cellulose Degrading Microorganism Using Genome Information 

From annotated sequences, the 16S rRNA gene sequence was identified and used for the preliminary identification of the bacteria isolated. The sequence was subjected to a BLASTN search against the NCBI database for bacteria. The highly similar sequences from the database were noted, and a library of the sequences from closely related organisms was created. Whole genome sequences for the closely related organisms from 16S rRNA alignment were retrieved from the NCBI database. Species level identification of the isolate was achieved using the online average nucleotide identity (ANI) calculator [19]. 

### 2.6. Analysis of Annotated Protein Sequences for Presence of Cellulose Operon Components

The genes from the whole genome of the bacterium were annotated, and genes associated with cellulose synthesis and degradation were searched for. The search terms included gene names such as endoglucanase, cellulase, cellobiose degraders, and endo-1,4-beta-D-glucanase. 

In addition, any unannotated genes adjacent to any significant hits were also analysed using the BLASTP search tool. If any new genes were identified, they were further investigated using online protein analysis tools to predict the conserved domains and their possible function. The intergenic sequences upstream to any new gene were analysed for the presence of ribosomal binding sites and transcriptional promoters using online bacterial promoter predictors (BPROM and PePPER) [20,21]. 

### 2.7. Cellulase Activity Assay

Cellulase activity was indicated by the growth of isolates on solid media containing cellulose as the sole carbon source. Further confirmation was noted by visualization of the zone of CMC degradation surrounding the colony or streak, which was further aided by flooding the solidified media with Gram’s iodine [14].

## 3. Results

Fresh kangaroo scats were collected each March from 2014 to 2020. Scats were homogenised in phosphate buffered saline (PBS), and aliquots of the suspensions were plated onto nutrient agar and solidified media containing CMC to find microbes capable of cellulose utilization. All incubations were aerobic at 37°C. Colonies were picked from CMC plates and subcultured to obtain pure isolates. MALDI-TOF mass spectrometry (Bruker Biotyper) analysis of freshly grown isolates revealed that close similarity to *Siccibacter colletis*: *Siccibacter turicensis* was not in the database that came with the biotyper. Colony PCR of the isolated colony was performed to obtain a 0.6 kb PCR product that was sequenced. The partial 16S rRNA gene sequence was used for preliminary identification. A microbe comprising ~5% of the population growing on CMC, designated #493, was subjected to further study, including whole genome sequence analysis. 

The whole genome sequence was 4.28 Mb in length with 4093 genes, among which 3944 were protein coding, 33 were pseudogenes, and 116 were RNA encoding genes. The GC content of the whole genome sequence was 58.1%. From the whole genome, the complete 16S rRNA gene sequence was obtained and analysed by using BLASTN searches in the NCBI database. The investigation showed the 16S rRNA gene sequence was most similar to the 16S rRNA gene sequence of *Cronobacter zurichensis* LMG 23730 (Updated Nomenclature: *Siccibacter turicensis*) (Table 1).

The whole genome sequence from #493 was compared with that of Type strains using the online ANI calculator from Kostas lab [19]. The #493 genome had the highest similarity to the genome of *Siccibacter turicensis* LMG 23730, evident by the ANI value of 97.98% (Table 2).

A search of the genes annotated using RAST identified nine genes that were involved in cellulose metabolism and a tenth gene with a possible role in cellulose metabolism (Table 3). 

**Figure 1:**
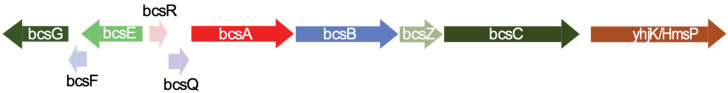
Schematic representation of genes involved in cellulose metabolism present in *S. turicensis*.

Based on the location and direction of the genes in the operon for cellulose metabolism, the cellulose operon present in the isolate is represented schematically in Figure 1, where we show the gene *yhjK* that might be involved in the cellulose metabolism. 

Investigation of the intergenic sequence between *bcsC* and *yhjK* using the BPROM online promoter prediction revealed the presence of a promoter region, −10 to −35 upstream of *yhjK*, while, another online tool, PePPER, did not predict any promoter region in the intergenic sequence. Furthermore, Shine-Dalgarno sequences were also identified 8 bases upstream of the start codon of the *yhjK* gene, which was complementary to a 3′ end of the 16S rRNA sequence. 

**Figure 2:**
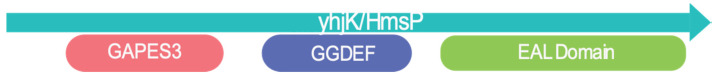
Domain analysis of the predicted YhjK/HmsP protein to demonstrate its relevance in cellulose operon.

Gene *yhjK* was translated using the Expasy Translate tool. The predicted protein was subjected to the conserved domain analysis, and three conserved domains were identified. These domains included a periplasmic sensor domain (GAPES3), cyclic diGMP cyclase domain (GGDEF), and an EAL domain (Figure 2). 

**Figure 3:**
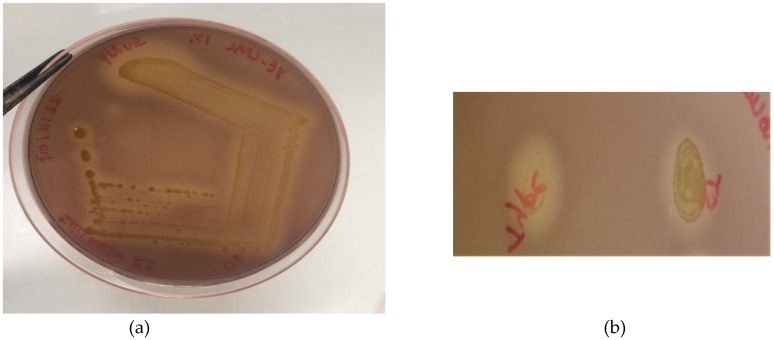
Cellulase activity assay in carboxymethyl cellulose (CMC) plates after Gram’s Iodine treatment. (**a**) zone of CMC clearance after streaking plating the #493 isolate. (**b**) similarity in cellulase activity between the *S. turicensis* Type strain (left) and isolate #493 (right).

Our study also involved cellulase activity assessment, however the cellulase activity of the bacterial isolates were too weak to be measured in culture supernatants and cleared lysates of sonicated cells. However, cellulose activity of #493 and *S. turicensis* DSM 18397 could be observed in the culture of the strains on CMC-containing plates, followed by staining with Gram’s iodine (Figure 3). 

## 4. Discussion

The diet of kangaroos consists exclusively of grasses, so gut microbes capable of cellulose degradation are expected to contribute to the nutrition of kangaroos [2]. In searching for cellulose-degrading microorganisms from kangaroos, the simple aerobic culture of microbes from kangaroo scats on selective media was performed. A frequently obtained isolate capable of degrading cellulose was *S. turicensis*, a member of the Enterobacteriaceae family. The Average Nucleotide Identity of our isolate with the *S. turicensis* Type strain was 97.98%, well above the species demarcation value of 95% [19]. *Siccibacter turicensis* was initially isolated in 2007, from fruit powder [22], and in 2018, from a patient with angular cheilitis [23], but has never been reported from scats of animals and has not been reported to degrade cellulose. So far, we have found it in all kangaroo scats that we sampled in Warrandyte, Victoria, Australia. It is of interest to investigate more widespread populations and other herbivores.

The nomenclature of the microorganism has undergone frequent re-evaluation with the initial name *Enterobacter turicensis* LMG 23730 changed to *Cronobacter zurichensis* LMG 23730, followed by a change to *Siccibacter turicensis* LMG 23730 [22,24,25]. The genomic analysis of the bacterium isolated from *S. turicensis* has revealed the presence of an operon for cellulose metabolism that is similar to that of *Salmonella enterica* serovar Typhimurium [26]. This study reports that *S. turicensis* isolated from kangaroo scats contains genes for the cellulose metabolism in the *bcs* operon. 

The genetic elements of the *bcs* operon were previously recognised in *Salmonella enteritidis* and *Salmonella enterica* serovar Typhimurium, *Pseudomonas putida*, *Burkholderia pseudomallei*, *Chromobacterium violaceum*, *Nostoc* spp., *Agrobacterium fabrum*, etc. [26,27]. From this study, genetic elements including *bcsEFG*, *bcsABZC*, and *bcsRQ* were identified in *S. turicensis*, which exactly matches the operon structure present in *S.* Typhimurium [13]. Previous studies, found that genes from *bcsAB* were involved in cellulose biosynthesis [28]. The gene *bcsC* is involved in helping the biosynthetic release of cellulose by encoding a protein that forms the pore in the outer membrane [13]. Recently, the *bcsG* gene has been characterised as encoding a Zn^2+^-dependent phosphoethanolamine transferase, which has a crucial role in cellulose formation and maintenance of biofilm integrity [29]. However, the function of the gene *bcsF* is not understood yet. Meanwhile, *bcsE* has a well-known function in production of the cyclic di-GMP binding protein, one of the major facilitators of cellulose biosynthesis [26]. The genes *bcsRQ* are involved in the regulation of cellulose biosynthesis. The majority of genes in the operon are involved in cellulose synthesis, however, the *bcsZ* gene downregulates the biosynthesis of cellulose and encodes a cellulase enzyme [30]. The biological role of the enzyme in microbes is to enhance the pathogenicity of the possessing organism by degrading the cellulose biofilm produced by the organism to survive the harsh environment, and thus free the organism to further spread [27]. 

Additionally, a gene which might be involved in cellulose metabolism was also found immediately downstream of *bcsC*. It is not only present in this organism, but was also found downstream of *bcsC* of *S.* Typhimurium. Investigation of the intergenic sequence for promoter sequences using the BPROM online tool revealed presence of a promoter, however, another bioinformatics tool, PePPER, did not predict a promoter within the sequence. The presence of a Shine-Dalgarno sequence suggests that the gene is likely to be translated to protein. Additionally, the downstream gene was analysed using multiple protein analysis tools to ascertain the type of protein it forms. BLASTP searches revealed its high similarity with the biofilm regulator *HmsP* gene for *S. turicensis*. The bioinformatics domain analysis using HMMER 3.2.1 revealed that the protein has three major parts: GAPES3 (GAmmaproteobacterial PEriplasmic Sensor), GGDEF, and EAL domain. Intracellular Cyclic diGMP, controlled by specific cyclic diGMP cyclase (with GGDEF domain) and phosphodiesterase (with EAL domain), acts as second messenger for activating genes responsible for biofilm formation [31]. Proteins with a GGDEF domain with N-terminus sensory functions have been found to activate the diguanylate cyclase activity on itself, which may independently limit the cellulose biosynthesis [32]. The GAPES3 domain has been reported to act as a periplasmic sensor responsible for negative regulation of biofilm formation in some pathogens like *Yersinia pestis* [33]. With these considerations, it is highly likely that this tenth gene is also involved in cellulose metabolism in *S. turicensis*, which may be regulating cellulose formation and degradation. Further study in determining the role of this gene in cellulose metabolism of *S. turicensis* may provide clarity.

The cellulase activity of #493 provided the justification for searching the genome to identify the presence of significant genes. There is tremendous potential for endoglucanases in industrial microbiology, food processing, feed improvement for domesticated animals, and bioremediation projects. On the contrary, the enzyme activity from *S. turicensis* was found to be weak as compared to the activity of the cellulases from *Bacillus amyloliquefaciens*, a well-known cellulose degrader (data not shown). It may be possible in the future to manipulate culture conditions to induce a higher expression of cellulase activity. The low activity of the cellulase in this organism could be partly due to its periplasmic localization, while *B. amyloliquefaciens* possesses a secreted protein. We suggest that *S. turicensis* enhances kangaroos’ survival in harsh environments by helping to digest the cellulosic diet. 

The beneficial roles of the cellulases, such as the endoglucanase encoded by organisms like *S. turicensis*, could potentially be exploited by using the organisms as a probiotic microbe for cattle feed, which may aid ruminant digestion of cellulose.

## 5. Conclusions

The facultative anaerobic Enterobactericeae family member *S. turicensis*, with cellulose degrading capacity, was isolated and identified from kangaroo scats. The whole genome sequence analysis of the isolate revealed the presence of the genes responsible for cellulose metabolism. This is an illustration of the importance of the gut microbes of kangaroos in providing nutrition and aiding survival. 

## Figures and Tables

**Table 1:** Identification of the cellulose degrader by pairwise alignment of the 16S rRNA sequence.

Highly Similar Organism (16S rRNA Sequences)	Raw Score	Query Cover (%)	Identity (%)	E-Value	NCBI Accession Number
*Cronobacter zurichensis* strain LMG 23730 (*Siccibacter turicensis*)	2627	98	99	0.0	NR_104924.1
*Erwinia tasmaniensis* strain Et1/99	2505	99	98	0.0	NR_074869.1
*Yokenella regensburgei* strain CIP 105435	2488	99	98	0.0	NR_104934.1
*Escherichia hermannii* strain CIP 103176	2475	99	98	0.0	NR_104940.1
*Pantoea agglomerans* strain NCTC9381	2471	99	97	0.0	NR_114735.1

**Table 2:** Average nucleotide identity of #493 isolate with two closely related organisms.

Whole Genome	Average Nucleotide Identity (ANI)	
	Two-Way ANI (%)	Standard Deviation
*Siccibacter turicensis* LMG 23730	97.98	1.41
*Siccibacter colletis*	84.99	4.26

**Table 3:** Predicted proteins of cellulose metabolism identified from the #493 genome sequences (annotated using RAST, Pfam, and BLAST).

Protein Names from RAST Annotation	Annotation from RAST Tool	Protein Family	Length (aa)	Most similar Protein in NCBI Database (BLASTP)	Accession Number
Prot_03025 scaffold_4_205556_203871	FIG002337: predicted inner membrane protein	BcsG	561	Cellulose biosynthesis protein BcsG [*S. turicensis*]	WP_106877578.1
Prot_03026 scaffold_4_205743_205549	Not annotated	BcsF	64	BcsF [*S. turicensis*]	WP_031521981.1
Prot_03027 scaffold_4_207095_205740	FIG005274: hypothetical protein	BcsE	415	Cellulose biosynthesis protein BcsE [*S. turicensis*]	WP_106877577.1
Prot_03028 scaffold_4_207471_207656	FIG004405: putative cytoplasmic protein	BcsR *	61	Hypothetical protein from Enterobacteriaceae	WP_024549966.1
Prot_03029 scaffold_4_207668_208402	Cellulose synthase, putative	BcsQ	244	Cellulose biosynthesis protein BcsQ [*S. turicensis*]	WP_106877576.1
Prot_03030 scaffold_4_208399_211017	UDP-forming cellulose synthase catalytic subunit	BcsA	872	UDP-forming cellulose synthase catalytic subunit [*S. turicensis*]	WP_106877575.1
Prot_03031 scaffold_4_211028_213472	Cyclic di-GMP binding protein precursor	BcsB	814	Cellulose biosynthesis cyclic di-GMP-binding regulatory protein BcsB [*S. turicensis*]	WP_106877574.1
Prot_03032 scaffold_4_213483_214586	Endo-1,4-β-D-glucanase	BcsZ	367	Endoglucanase [*S. turicensis*]	WP_106877573.1
Prot_03033 scaffold_4_214568_218068	Cellulose synthase operon protein C	BcsC	1166	Cellulose biosynthesis protein BcsC [*S. turicensis*]	WP_106877572.1
Prot_03034 scaffold_4_218174_220189	Protein yhjK	GAPES3/GGDEF/EAL	671	Biofilm formation regulator HmsP [*S. turicensis*]	WP_106877571.1

* BcsR has not been reported in *S. turicensis* in previous annotations.

## References

[1] Barker C.J., Gillett A., Polkinghorne A., Timms P. (2013). Investigation of the koala (*Phascolarctos cinereus*) hindgut microbiome via 16S pyrosequencing. Vet. Microbiol..

[2] Arman S.D., Prideaux G.J. (2015). Dietary classification of extant kangaroos and their relatives (Marsupialia: Macropodoidea). Austral Ecol..

[3] Meng F., Ma L., Ji S., Yang W., Cao B. (2014). Isolation and characterization of *Bacillus subtilis* strain BY-3, a thermophilic and efficient cellulase-producing bacterium on untreated plant biomass. Lett. Appl. Microbiol..

[4] Poomai N., Siripornadulsil W., Siripornadulsil S. (2014). Cellulase enzyme production from agricultural waste by *Acinetobacter* sp. KKU44. Adv. Mater. Res..

[5] Wang D., Wang Z., He F., Kinchla A.J., Nugen S.R. (2016). Enzymatic digestion for improved bacteria separation from leafy green vegetables. J. Food Prot..

[6] Wang H., Wang H., Xing T., Wu N., Xu X., Zhou G. (2016). Removal of *Salmonella* biofilm formed under meat processing environment by surfactant in combination with bio-enzyme. LWT-Food Sci. Technol..

[7] Zulkarnain D., Zuprizal Z., Wihandoyo W., Supadmo S. (2016). Effect of cellulase supplementation on in vitro digestibility and energy, crude fiber and cellulose content of sago palm (*Metroxylon* sp.) waste as broiler chicken feed. Pak. J. Nutr..

[8] Ghodadara S., Shilpkar P., Dungrechiya A. (2015). Optimized production of cellulase by *Aspergillus niger* using ricinus communis seed coat waste. J. Pure Appl. Microbiol..

[9] Khan M.N., Luna I.Z., Islam M.M., Sharmeen S., Salem K.S., Rashid T.U., Zaman A., Haque P., Rahman M.M. (2016). Cellulase in Waste Management Applications. New and Future Developments in Microbial Biotechnology and Bioengineering: Microbial Cellulase System Properties and Applications.

[10] Pawar K.D., Dar M.A., Rajput B.P., Kulkarni G.J. (2014). Enrichment and identification of cellulolytic bacteria from the gastrointestinal tract of giant african snail, *Achatina fulica*. Appl. Biochem. Biotechnol..

[11] Thongekkaew J., Patangtasa W., Jansri A. (2014). Cellulase and xylanase production from *Candida easanensis* using agricultural wastes as a substrate. Songklanakarin J. Sci. Technol..

[12] Wong H.C., Fear A.L., Calhoon R.D., Eichinger G.H., Mayer R., Amikam D., Benziman M., Gelfand D.H., Meade J.H., Emerick A.W. (1990). Genetic organization of the cellulose synthase operon in *Acetobacter xylinum*. Proc. Natl. Acad. Sci. USA.

[13] Römling U., Galperin M.Y. (2015). Bacterial cellulose biosynthesis: Diversity of operons, subunits, products, and functions. Trends Microbiol..

[14] Kasana R.C., Salwan R., Dhar H., Dutt S., Gulati A. (2008). A rapid and easy method for the detection of microbial cellulases on agar plates using Gram’s iodine. Curr. Microbiol..

[15] Boath J.M., Dakhal S., Van T.T.H., Moore R.J., Dekiwadia C., Macreadie I.G. (2020). Polyphasic characterisation of *Cedecea colo* sp. Nov., a new enteric bacterium isolated from the koala hindgut. Microorganisms.

[16] Baker G.C., Smith J.J., Cowan D.A. (2003). Review and re-analysis of domain-specific 16S primers. J. Microbiol. Methods.

[17] Coil D., Jospin G., Darling A.E. (2015). A5-miseq: An updated pipeline to assemble microbial genomes from Illumina MiSeq data. Bioinformatics (Oxf. Engl.).

[18] Aziz R.K., Bartels D., Best A., DeJongh M., Disz T., Edwards R.A., Formsma K., Gerdes S., Glass E.M., Kubal M. (2008). The RAST Server: Rapid annotations using subsystems technology. BMC Genom..

[19] Goris J., Konstantinidis K.T., Klappenbach J.A., Coenye T., Vandamme P., Tiedje J.M. (2007). DNA-DNA hybridization values and their relationship to whole-genome sequence similarities. Int. J. Syst. Evol. Microbiol..

[20] de Jong A., Pietersma H., Cordes M., Kuipers O.P., Kok J. (2012). PePPER: A webserver for prediction of prokaryote promoter elements and regulons. BMC Genom..

[21] Solovyev V., Salamov A. (2011). Automatic annotation of microbial genomes and metagenomic sequences. Metagenomics and Its Applications in Agriculture, Biomedicine and Environmental Studies.

[22] Stephan R., Van Trappen S., Cleenwerck I., Vancanneyt M., De Vos P., Lehner A. (2007). *Enterobacter turicensis* sp. nov. and *Enterobacter helveticus* sp. nov., isolated from fruit powder. Int. J. Syst. Evol. Microbiol..

[23] Lepuschitz S., Pekard-Amenitsch S., Haunold R., Schill S., Schriebl A., Mach R., Allerberger F., Ruppitsch W., Forsythe S.J. (2018). Draft genome sequence of the first documented clinical *Siccibacter turicensis* isolate in Austria. Genome Announc..

[24] Jackson E.E., Sonbol H., Masood N., Forsythe S.J. (2014). Genotypic and phenotypic characteristics of *Cronobacter species*, with particular attention to the newly reclassified species *Cronobacter helveticus, Cronobacter pulveris*, and *Cronobacter zurichensis*. Food Microbiol..

[25] Stephan R., Grim C.J., Gopinath G.R., Mammel M.K., Sathyamoorthy V., Trach L.H., Chase H.R., Fanning S., Tall B.D. (2014). Re-examination of the taxonomic status of *Enterobacter helveticus*, *Enterobacter pulveris* and *Enterobacter turicensis* as members of the genus *Cronobacter* and their reclassification in the genera *Franconibacter* gen. nov. and *Siccibacter* gen. nov. as *Franconibacter helveticus* comb. nov., *Franconibacter pulveris* comb. nov. and *Siccibacter turicensis* comb. nov., respectively. Int. J. Syst. Evol. Microbiol..

[26] Ahmad I., Rouf S.F., Sun L., Cimdins A., Shafeeq S., Guyon S., Schottkowski M., Rhen M., Römling U. (2016). BcsZ inhibits biofilm phenotypes and promotes virulence by blocking cellulose production in *Salmonella enterica* serovar Typhimurium. Microb. Cell Factories.

[27] Solano C., García B., Valle J., Berasain C., Ghigo J.M., Gamazo C., Lasa I. (2002). Genetic analysis of *Salmonella enteritidis* biofilm formation: Critical role of cellulose. Mol. Microbiol..

[28] Morgan J.L.W., Strumillo J., Zimmer J. (2013). Crystallographic snapshot of cellulose synthesis and membrane translocation. Nature.

[29] Sun L., Vella P., Schnell R., Polyakova A., Bourenkov G., Li F., Cimdins A., Schneider T.R., Lindqvist Y., Galperin M.Y. (2018). Structural and Functional Characterization of the BcsG Subunit of the Cellulose Synthase in *Salmonella* Typhimurium. J. Mol. Biol..

[30] Yeoman C.J., Han Y., Dodd D., Schroeder C.M., Mackie R.I., Cann I.K.O. (2010). Chapter 1-Thermostable Enzymes as Biocatalysts in the Biofuel Industry. Advances in Applied Microbiology.

[31] Ryjenkov D.A., Tarutina M., Moskvin O.V., Gomelsky M. (2005). Cyclic diguanylate is a ubiquitous signaling molecule in bacteria: Insights into biochemistry of the GGDEF protein domain. J. Bacteriol..

[32] Sarenko O., Klauck G., Wilke F.M., Pfiffer V., Richter A.M., Herbst S., Kaever V., Hengge R. (2017). More than Enzymes That Make or Break Cyclic Di-GMP—Local Signaling in the Interactome of GGDEF/EAL Domain Proteins of *Escherichia coli*. mBio.

[33] Bobrov A.G., Kirillina O., Perry R.D. (2005). The phosphodiesterase activity of the HmsP EAL domain is required for negative regulation of biofilm formation in *Yersinia Pestis*. FEMS Microbiol. Lett..

